# Genetic Profile of Adenomatoid Odontogenic Tumor and Ameloblastoma. A Systematic Review

**DOI:** 10.3389/froh.2021.767474

**Published:** 2021-11-15

**Authors:** Constanza Marín, Sven E. Niklander, René Martínez-Flores

**Affiliations:** ^1^Unidad de Patología y Medicina Oral, Facultad de Odontología, Universidad Andres Bello, Viña del Mar, Chile; ^2^Unit of Oral and Maxillofacial Medicine, Pathology and Surgery, University of Sheffield, Sheffield, United Kingdom

**Keywords:** odontogenic tumors, adenomatoid odontogenic tumor, amelobalstoma, genetic mutation, BRAF, KRAS

## Abstract

**Purpose:** To perform a comprehensive and systematic critical appraisal of the genetic alterations reported to be present in adenomatoid odontogenic tumor (AOT) compared to ameloblastoma (AM), to aid in the understanding in their development and different behavior.

**Methods:** An electronic search was conducted in PubMed, Scopus, and Web of Science during March 2021. Eligibility criteria included publications on humans which included genetic analysis of AOT or AM.

**Results:** A total of 43 articles reporting 59 AOTs and 680 AMs were included. Different genomic techniques were used, including whole-exome sequencing, direct sequencing, targeted next-generation sequencing panels and TaqMan allele-specific qPCR. Somatic mutations affecting *KRAS* were identified in 75.9% of all AOTs, mainly G12V; whereas a 71% of the AMs harbored *BRAF* mutations, mainly V600E.

**Conclusions:** The available genetic data reports that AOTs and AM harbor somatic mutations in well-known oncogenes, being KRAS G12V/R and BRAFV600E mutations the most common, respectively. The relatively high frequency of ameloblastoma compared to other odontogenic tumors, such as AOT, has facilitated the performance of different sequencing techniques, allowing the discovery of different mutational signatures. On the contrary, the low frequency of AOTs is an important limitation for this. The number of studies that have a assessed the genetic landscape of AOT is still very limited, not providing enough evidence to draw a conclusion regarding the relationship between the genomic alterations and its clinical behavior. Thus, the presence of other mutational signatures with clinical impact, co-occurring with background *KRAS* mutations or in wild-type *KRAS* cases, cannot be ruled out. Since BRAF and RAS are in the same MAPK pathway, it is interesting that ameloblastomas, frequently associated with BRAFV600E mutation have aggressive clinical behavior, but in contrast, AOTs, frequently associated with RAS mutations have indolent behavior. Functional studies might be required to solve this question.

## Introduction

Adenomatoid Odontogenic Tumor (AOT) and Ameloblastoma (AM) are benign epithelial odontogenic tumors affecting most commonly the tooth bearing areas of the jaws. Both tumors are composed of a proliferation of epithelial cells arranged in a way that reminds to some extent, to the enamel organ of a tooth germ [1]. AM is well-known for being locally infiltrating, for its continuous growth, its high rates of recurrences if not adequately removed and the possibility of undergoing malignant transformation [2]. On the contrary, AOT manifests clinically as a slow and self-limiting growth which does not require the aggressive surgical approach usually adopted for AM. In AOT, recurrences are extremely rare, even if it is partially removed [3]. The clinicopathological features of both tumors, allows to consider AM as a neoplasm, whereas there is a general agreement that AOT may represent a hamartoma [3–5], although this is a matter of debate.

Different genomic alterations, which includes chromosomal imbalances and genetic mutations, have been reported to be present in ameloblastomas. Mutations in genes that belong to the mitogen-activated protein kinase (MAPK) pathway are present in almost 90% of ameloblastomas, with BRAF V600E, being the most described mutation [6–10]. The prevalence of BRAF V600E in ameloblastoma ranges from 46 [6] to 90% [11], with a mean value of 68%. Other somatic mutations have been reported, either in MAPK or non-MAPK pathways [6, 8, 10, 12–16]. Some of these, such as mutations in *PTEN, SMARCB1, EGFR, TP53, CTNNB1*, and *PIK3CA* [6, 8–10], can occur in the background of the classical BRAF V600E mutation. Nevertheless, mutations in *SMO, FGFR2, KRAS, HRAS*, and *NRAS* have been reported to be mutually exclusive with BRAF V600E [8, 14]. Moreover, deletions in chromosome 22 [6, 17–19] and copy number alterations in *BAG1, PPP2R5A*, and *PKD1L2* [20] have also been reported and could also be involved in the pathogenesis of the tumor.

Little information is known about the genetic background of AOT. Mutations in the β-catenin gene (*CTNNB1*) have been suggested, as strong cytoplasmatic expression of β-catenin is reported using immunohistochemistry [4, 21]. However, authors have failed to show alterations in *CTNNB1* [4]. Nevertheless, other more recent studies have shown consistent mutations in *KRAS* [22–24] and copy number alterations [23] affecting *IGF2BP3*.

As both AM and AOT have different clinical behavior, suggesting a different biological nature, and there has been a significant interest in papers reporting their genetic alterations during the last years, the aim of this systematic review was to compare the genetic alterations of AOT with the ones reported in AM, in order to summarize the current genetic knowledge of these lesions and aid in the understanding of the genomic alterations underlying their development.

## Methods

This systematic review was conducted following the PRISMA Statement guidelines.

### Eligibility Criteria

Parameters were kept broad to maximize search results. The inclusion criteria consisted of full text observational research studies on humans about genetic analysis of adenomatoid odontogenic tumor or ameloblastoma, with or without clinicopathological and treatment information. Studies were excluded if they were about polymorphisms, were performed *in-vitro* or were not performed on human participants. Conference abstracts, articles where the full text was unavailable, bioinformatic research only, reviews, case reports, or case series without genetic analysis were also excluded.

### Information Sources and Search Strategy

A preliminary literature search was conducted by one of the authors (RMF) to guide the search strategy. The search was conducted in PubMed, Scopus, and Web of Science during March of 2021, restricted to human studies in English language and without year restrictions. The following keywords were used in the identification of potential articles: (adenomatoid odontogenic tumor OR ameloblastoma) AND (chromosomal alteration OR copy number variation OR deletion OR gene mutation OR genetic OR genomic OR genome OR insertion OR loss of heterozygosity OR microarray OR sanger sequencing OR single nucleotide variant OR targeted next-generation sequencing OR whole exome sequencing). This was further complemented with manual searches using the reference list of each identified study.

### Selection Process

**Figure 1:**
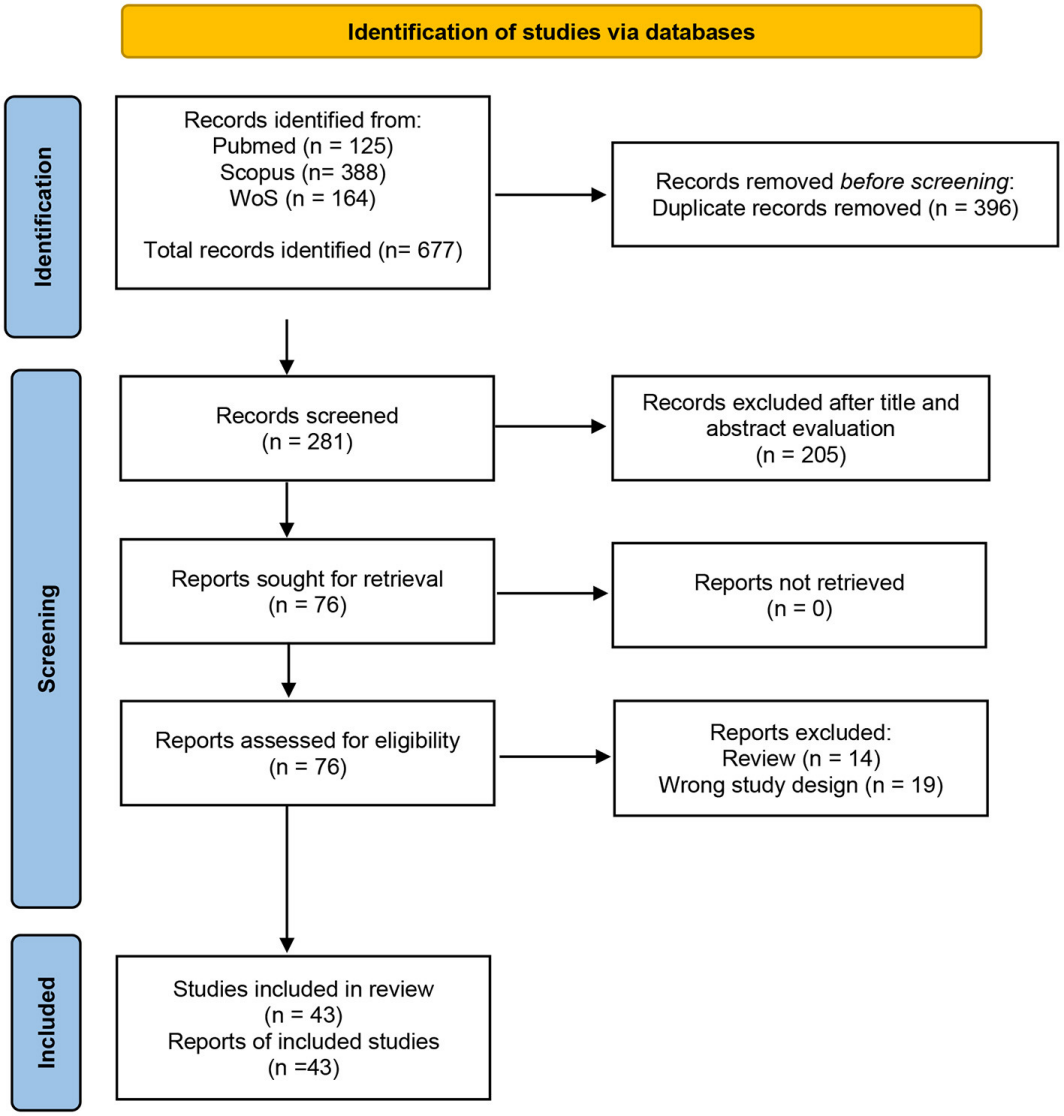
PRISMA flow diagram.

After the removal of duplicates, two independent researchers (SN and CM) read the title and abstract to identify and select articles. Full text of selected studies were then analyzed and those who met the eligibility criteria were included in the review (Figure 1). Any disagreement was resolved through discussion guided by a third researcher who acted as a referee (RMF). No automation tools were used in this process

### Data Collection Process

After reading the full text, two independent researchers (SN and CM) extracted and transferred the data to a Microsoft Excel spreadsheet (Microsoft Office 365^®^). Any disagreement was resolved through discussion guided by a third researcher who acted as a referee (RMF). No automation tools were used in this process.

### Study Risk of Bias Assessment

The entities included in this review are rare and as such, the highest quality of primary data is from case series. At present, there is no agreed guidelines to perform and report molecular biology studies about odontogenic tumors. Hence, there is substantial heterogeneity in their data recording and reporting. Given these limitations, the risk of bias will be uncertain on almost all reported case series, with low quality of evidence. Therefore, we have decided not to undertake these assessments.

### Synthesis Methods

A narrative synthesis of the data is planned. The characteristics collected from the studies to do the quantitative analysis will be based on: first author, year, country, tissue sample, sample size, gene or chromosome involved, gene mutation, signaling pathway, and genetic assay. Visualization of the data will be presented in form of figures and tables.

## Results

The search results are outlined in a PRISMA flow diagram in Figure 1. The initial literature search identified 677 studies. Following duplicate removal (*n* = 396), 281 studies had their titles and abstracts screened by two of the reviewers (SN and CM) from which 205 articles were removed. Finally, 76 studies were included for full text evaluation to ensure they satisfied the inclusion and exclusion criteria. Thirty-four articles were excluded with the following reasons: reviews (*n* = 14) and wrong study design (*n* = 19). In total, 43 articles were included in this systematic review (Figure 1).

### Adenomatoid Odontogenic Tumor

#### Gene Mutations

**Figure 2:**
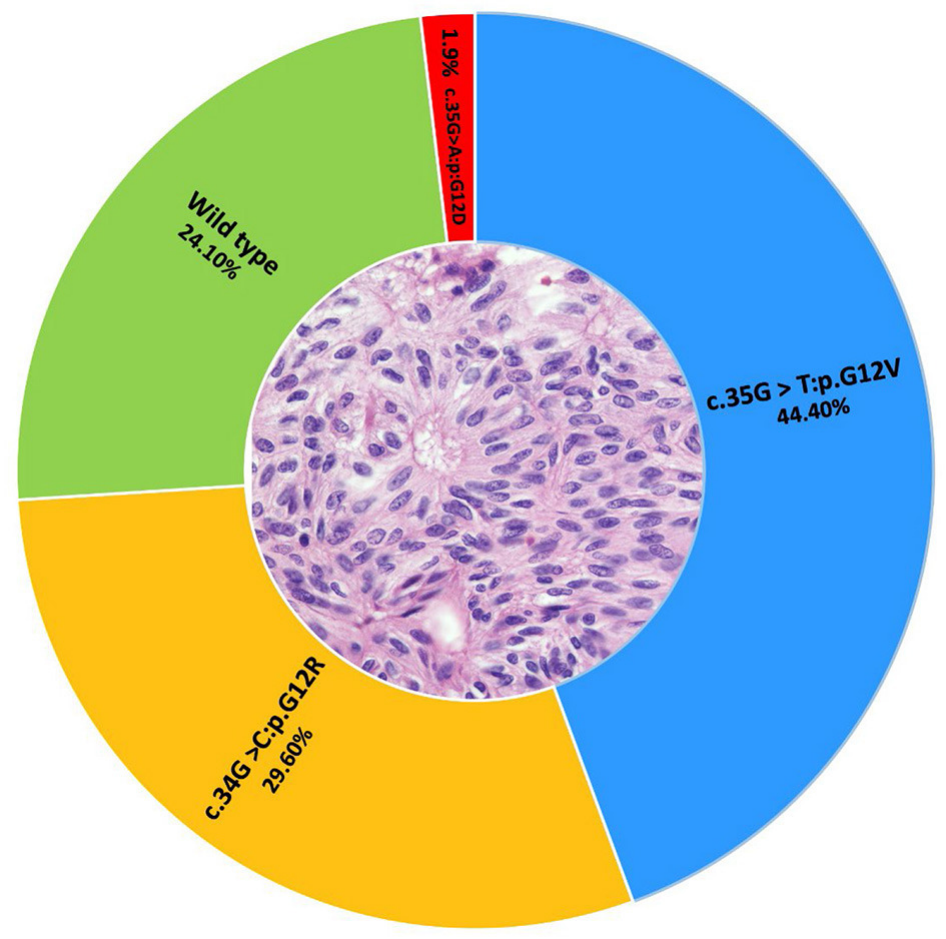
A total of 54 AOTs were assessed for *KRAS* mutations. The KRAS G12V mutation was identified in 24 cases, G12R in 16 cases and G12D in one tumor. The remaining 13 cases corresponded to wild type cases.

**Figure 3:**
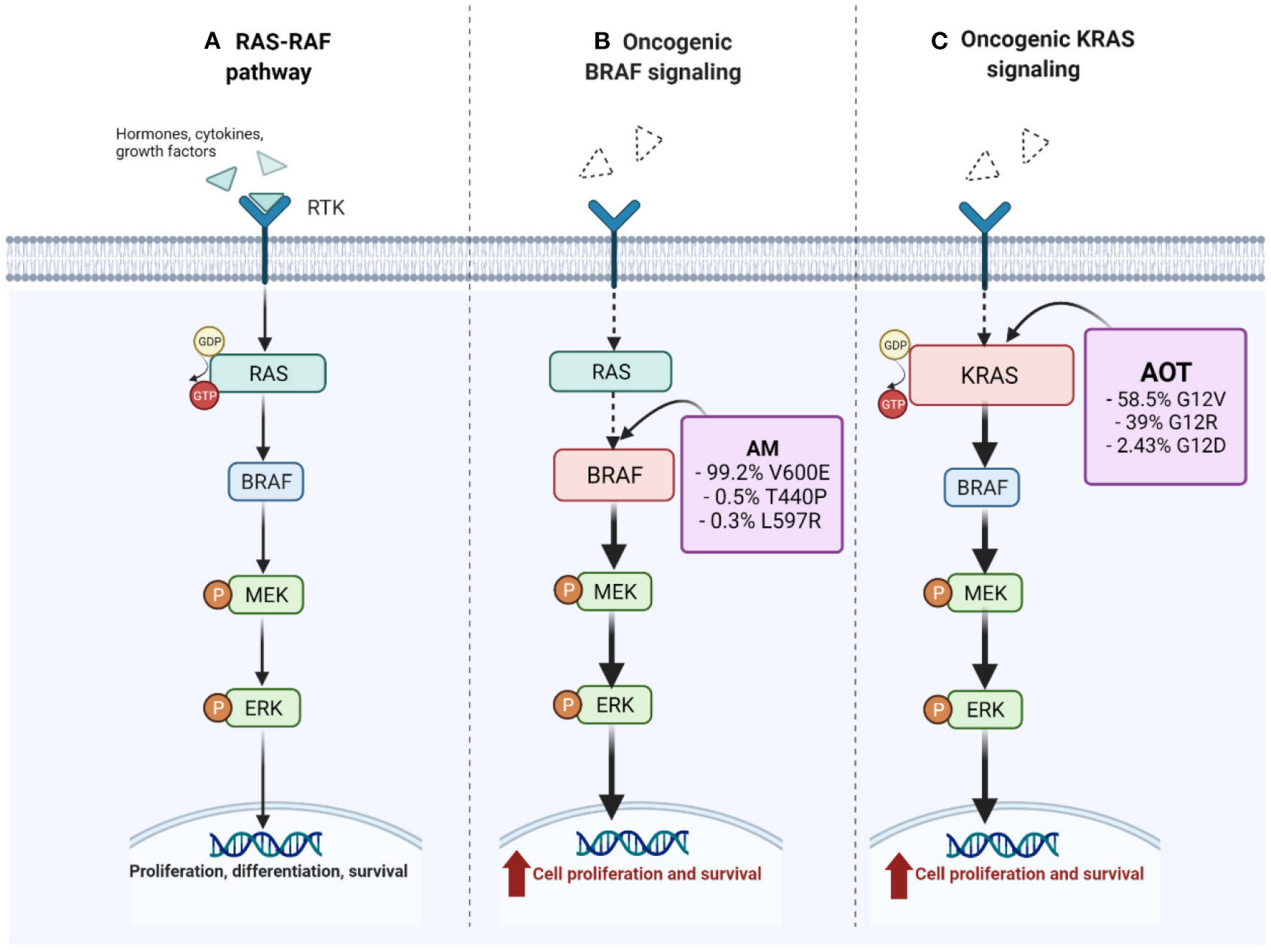
Constitutive and oncogenic activation of MAPK/ERK pathway. In **(A)**, several growth factors, hormones, and cytokines activate the receptor tyrosine-kinase (RTK) favoring the constitutive activation of RAS by switching GDP-GTP toward the activate state. The downstream signaling is regulated by RAS-GTP and additional proteins that are not shown in this figure. Ras activates BRAF which facilitates the phosphorylation of MEK, which in turn allows the phosphorylation and activation of ERK. The resulting signaling cascade culminates with translocation of ERK to the nucleus, and the activation of transcription factors that result in the expression of genes related to proliferation, differentiation, and survival. In **(B,C)**, in the presence of oncogenic BRAF and KRAS, respectively, the constitutive activation is independent of extracellular factors and does not respond to biochemical signals that would normally regulate the activity. Adapted from “Vemurafenib in Oncogenic BRAF Signaling Pathway in Melanoma,” by BioRender.com (2021). Retrieved from: https://app.Biorender.com/biorender-templates.

Six articles reported mutations in AOT [4, 22–26]. A total of 59 AOTs were assessed under different genomic techniques, such as direct sequencing, targeted NGS panels, and TaqMan allele-specific qPCR (Table 1). *KRAS* was the most commonly affected gene among the studies that included this gene in their analysis. One article used TaqMan allele-specific qPCR for *KRAS* [22], whereas other two worked with a Targeted NGS panel which included RAS family [23, 24] (Table 1). A total of 54 samples were analyzed among these 3 studies, from which 75.9% harbored somatic mutations in *KRAS* (*n* = 41). All the studies reported that the mutations affecting *KRAS* corresponded to single nucleotide variations corresponding to missense mutations [22–24]. All the mutations affected codon 12, in which three types of transversions were identified: guanine to thymidine (G>T) in 24/41 cases, guanine to cytosine (G>C) in 16/41 cases and guanine to an adenosine (G>A) in one case. The aforementioned single base variations lead to G12V, G12R, or G12D substitutions, respectively [22–24] (Figures 2, 3).

**Table 1:** Gene mutations reported in AOT.

**References**	**Year**	**Country**	**Tissue sample**	**Sample size**	**Gene involved (*n*)**	**Gene mutation**	**Signaling pathway**	**Genetic technique assay**
Shimura et al. [25]	2020	Japan	FFPE, FT	1	*SMO (1)*	Y399S and Y394S	Hedgehog	Targeted NGS panel
Coura et al. [22]	2019	Brazil	FFPE	38	*KRAS (27)*	G12V (*n* = 15)	MAPK/ERK	TaqMan allele-specific qPCR, histological and morphometric analysis, immunohistochemistry and Sanger sequencing
						G12R (*n* = 12)		
						Wild type (*n* = 11)		
Bologna-Molina et al. [24]	2018	Japan	FFPE	9	*KRAS (7)*	G12D (*n* = 1)	MAPK/ERK	Targeted NGS, Luminex assay, and immunohistochemistry
						c.35G>T: p: G12V (*n* = 2)		
						G12R (*n* = 4)		
						Not suitable for analysis (*n* = 2)		
Gomes et al. [23]	2016	Brazil	N/A	9	*KRAS (7)*	G12	MAPK/ERK	Targeted NGS, Sanger sequencing, and qPCR
						Wild type (*n* = 2)		
Harnet et al. [4]	2013	France	FFPE	1	*CTNNB1*	No mutation	Wnt/β-catenin	Direct sequencing, immunohistochemistry
Perdigão et al. [26]	2004	Brazil	FFPE, FT	1	*AMBN (1)*	R90W	N/A	Direct sequencing

*N/A, Not Available*.

Among the remaining three studies, one article worked with a Targeted NGS panel assessing specifically mutations in *SMO, BRAF, PTCH1*, and *GNAS* [25]. Only one AOT was included and showed two missense mutations in *SMO* (Y394S and p.Y399S). No mutations affecting *BRAF* or *PTCH1* were reported [25].

By direct sequencing, one study identified one heterozygous missense mutation affecting *AMBN*, leading to a R90W substitution [26]. Using pyrosequencing and direct sequencing, Harnet et al., did not detect mutations affecting *CTNNB1* [4] in a follicular type of AOT (Table 1).

#### Chromosomal Alterations

To date there is only one published article about chromosomal alterations in AOT [23]. By using a whole-genome array and comparing those results with databases, Gomes et al. reported two rare losses in AOTs. One deletion affecting *IGF2BP3* at 7p15.3 in a single AOT, and another affecting the chromosome 6 at q15, but no gene was identified at that position. The deletion in *IGF2BP3* involved an intronic region of the protein-coding transcript, however, *in silico* analysis predicted the implication of the first exon of four alternative transcripts. Its potential in tumorigenesis remains unclear [23] (Table 2).

**Table 2:** Chromosomal alterations in AOT and AM.

**References**	**Year**	**Country**	**Tumor**	**Tissue sample**	**Sample size**	**Genetic technique assay**	**Chromosome**	**Alteration**	**Genes**
Diniz et al. [20]	2017	Brazil	AM, AC	FT	8, 1	Whole genome microarray, qPCR, and RT-qPCR	9p21.1	CNA Gain	B4GALT1 and BAG1
							16q23.2	CNA Loss	PKD1L2
							1q32.3	CNA Gain	PPP2R5A
Gomes et al. [23]	2016	Brazil	AOT	N/A	2	Whole genome microarray, targeted NGS, Sanger sequencing, and qPCR	7p15.3	CNA Loss	IGF2BP3
Toida et al. [19]	2005	Japan	AM	FT	9	Comparative genomic hybridization and FISH	1q	CNA Gain	N/A
							1pter, 10q, and 22q	CNA Loss	Potential candidate genes RIZ1 (1p36.3–p36.2), NBL1 (1p36.13–p36.11), TP73 (1p36.3), and CDC2L2 (1p36.3)
Nodit et al. [27]	2004	United States	AM, AC	FFPE	12, 3	Panel of microsatellite markers	1p34.2 and 10q23	Allelic loss	L- myc and PTEN
Jääskeläinen et al. [18]	2002	Finland	AM	FFPE	20	Comparative genomic hybridization and immunocytochemistry	21; 16q, 19p, and of 22	CNA Loss	N/A
							16p	CNA Gain	N/A
Guan et al. [13]	2019	Singapore	AM	FT	10	Whole-exome sequencing	None	None	N/A

*N/A, Not Available*.

### Ameloblastoma

#### Gene Mutations

Our search yielded 37 articles about the molecular landscape of ameloblastoma. A total of 680 tumors were assessed using small-to large-scale and “omics” techniques. Two studies performed whole-exome sequencing (WES) [13, 15], eight used targeted NGS panels [6, 8, 10, 12, 14, 16, 20, 25], and the remaining 26 performed either TaqMan-allele specific probes or direct sequencing (Table 3).

**Table 3:** Gene mutations reported in ameloblastoma.

**References**	**Year**	**Country**	**Tissue sample**	**Number of AM**	**Gene involved (*n*)**	**Gene mutation**	**Signaling pathway**	**Genetic technique assay**
Shi et al. [15]	2021	China	FT	4	*BRAF (4)*	V600E	MAPK/ERK	Whole exome sequencing
					*HSPA4 (2)*	E700G and H205N	N/A	
					*KMT2D (1)*	Frameshift deletion	N/A	
Shimura et al. [25]	2020	Japan	FFPE, FT	6	*BRAF (2)*	T440P	MAPK/ERK	Targeted NGS panel
					*PTCH1 (1)*	V582G	Hedgehog	
Derakhshan et al. [28]	2020	Iran	FFPE	50	*BRAF (46)*	V600E	MAPK/ERK	qRT-PCR, immunohistochemistry
Oh et al. [11]	2020	Korea	FFPE	28	*BRAF (24)*	V600E	MAPK/ERK	Sanger sequencing and immunohistochemistry
Sant'Ana et al. [29]	2020	Brazil	FFPE	5	*BRAF (4)*	V600E	MAPK/ERK	Taqman allele-specific qPCR, Sanger sequencing
Seki-Soda et al. [30]	2020	Japan	FFPE	21	*BRAF (16)*	V600E	MAPK/ERK	Sanger sequencing and immunohistochemistry
Zhang et al. [31]	2020	China	FFPE, FT	17	*BRAF (14)*	V600E	MAPK/ERK	Direct sequencing
Duarte-Andrade et al. [32]	2019	Brazil	FFPE	12	*BRAF (9)*	V600E	MAPK/ERK	Metabolic profiling by GC-MS and TaqMan allele-specific qPCR
Guan et al. [13]	2019	Singapore	FT	10	*BRAF (8)*	V600E	MAPK/ERK	Whole exome sequencing
					*ANKRD31 (2)*	P1580Q and D796Y	N/A	
					*CDC73 (2)*	L404I and P351T	N/A	
					*CREBBP (2)*	Frameshift deletion	N/A	
					*DHX29 (2)*	(G1121T and W374L); (L610F)	N/A	
					*KMT2D (2)*	Stop gain	N/A	
					*PLEKHN1 (2)*	Frameshift deletion	N/A	
					*BCOR (1)*	Frameshift deletion	N/A	
					*CTNNB1 (1)*	G34V and G27V	N/A	
					*LRP6 (1)*	P455S	N/A	
					*LAMB1 (1)*	Frameshift deletion	N/A	
					*SCN5A (1)*	F1908L; F1872L; F1925L; F1926L; F1893L	N/A	
Oh et al. [33]	2019	Korea	FFPE	30	*BRAF (27)*	V600E	MAPK/ERK	Sanger sequencing and immunohistochemistry
Narayan et al. [34]	2019	India	FFPE	20	*PTEN (5)*	V158E	PI3K/Akt/mTOR	Sanger sequencing
					*PTEN (5)*		PI3K/Akt/mTOR	
					*PTEN (5)*	Stop gain	PI3K/Akt/mTOR	
Xia et al. [35]	2019	China	FFPE	5	*BRAF (3)*	V600E	MAPK/ERK	TaqMan allele-specific qPCR, FISH, Alcian blue staining
Bartels et al. [12]	2018	Germany	FFPE	20	*BRAF (5)*	V600E	MAPK/ERK	Targeted NGS panel, FISH, immunohistochemistry, and pyrosequencing
				7	*FGFR2 (4)*	C383R (2)	FGF/FGFR	
					*FGFR2 (4)*	Y376C	FGF/FGFR	
					*FGFR2 (4)*	V396D	FGF/FGFR	
					*TP53 (1)*	R248Q	p53	
					*PTEN (1)*	Q171K	PI3K/Akt/mTOR	
					*KRAS (1)*	L56_G60dup	MAPK/ERK	
Gültekin et al. [10]	2018	France	FFPE	62	*SMO (8)*	L412F (6)	Hedgehog	Sanger sequencing
					*SMO (8)*	W535L (2)	Hedgehog	
					*BRAF (34)*	V600E	MAPK/ERK	Targeted NGS panel
					*NRAS (2)*	N/A	MAPK/ERK	
		Germany			*HRAS (1)*	N/A	MAPK/ERK	
					*EGFR (1)*	N/A	EGFR	
					*KRAS (2)*	N/A	MAPK/ERK	
					*PIK3CA (4)*	N/A	PI3K/AKT/mTOR	
		Turkey			*PTEN (2)*	N/A	PI3K/Akt/mTOR	
					*FGFR (1)*	N/A	FGF/FGFR	
					*CDKN2A (2)*	N/A	NS	
					*CTNNB1 (1)*	N/A	Wnt/β-catenin	
Heikinheimo et al. [14]	2018	Finland	FFPE, FT	73	*BRAF (58)*	V600E	MAPK/ERK	Targeted NGS panel, Sanger sequencing RT-qPCR and immunohistochemistry
					*SMO (1)*	L412F	Hedgehog	
					*FGFR2 (2)*	C382R	FGF/FGFR	
					*HRAS (2)*	Q61R	MAPK/ERK	
					*NRAS (2)*	Q61R	MAPK/ERK	
Soltani et al. [36]	2018	Iran	FFPE	19	*BRAF (12)*	V600E	MAPK/ERK	Direct sequencing
Diniz et al. [20]	2017	Brazil	FT	8	*BRAF (7)*	V600E	MAPK/ERK	Whole genome microarray, qPCR, and RT-qPCR
Yukimuri et al. [16]	2017	Japan	FFPE	14	*CTNNB1 (2)*	S37C and G34E	Wnt/β-catenin	Targeted NGS panel, Sanger sequencing, immunohistochemistry, immunocytochemistry, western blotting, cell culture.
					*NRAS (2)*	Q61R	MAPK/ERK	
					*BRAF (12)*	V600E	MAPK/ERK	
Li et al. [37]	2016	China	FT	30	*APC (ND)*	N/A	Wnt/β-catenin	Direct sequencing, Methylation detection of APC gene
Pereira et al. [38]	2016	Brazil	FFPE	8	*BRAF (5)*	V600E	MAPK/ERK	TaqMan allele-specific qPCR, Sanger sequencing and immunohistochemistry
Brunner et al. [39]	2015	Switzerland	FFPE	19	*BRAF (14)*	V600E	MAPK/ERK	Multiplex and nested PCR, Sanger sequencing, and FISH
Diniz et al. [9]	2015	Brazil	FFPE	17	*BRAF (14)*	V600E	MAPK/ERK	qPCR and Sanger sequencing
Brown et al. [8]	2014	United States	FFPE	50	*BRAF (31)*	V600E	MAPK/ERK	Allele-specific PCR, targeted NGS panel, Sanger sequencing, immunohistochemistry, western blotting, cell culture, and proliferation assays
					*KRAS (4)*	G12R	MAPK/ERK	
					*NRAS (3)*	Q61R (2) and Q61K (1)	MAPK/ERK	
					*HRAS (3)*	G12S, Q61R, Q61K	MAPK/ERK	
					*FGFR2 (3)*	C382R (2) and V395D	FGF/FGFR	
					*SMO (8)*	L412F (4)	Hedgehog	
					*SMO (8)*	W535L (3)	Hedgehog	
					*SMO (8)*	G416E (1)	Hedgehog	
					*CTNNB1 (2)*	S33P and S45P	Wnt/β-catenin	
					*PIK3CA (3)*	E542K, E545K, H1047R	PI3K/AKT/mTOR	
					*SMARCB1 (3)*	R77H	NS	
Kurppa et al. [7]	2014	Finland	FT	24	*BRAF (15)*	V600E	MAPK/ERK	Sanger sequencing, RT-qPCR, immunohistochemistry, western blotting, cell culture, and MTT cell viability assay
Li et al. [40]	2014	China	FFPE	20	*TSC1 (10)*	D24E; A84T; E445E; Q792R; C803S; L861L; Q990Q	mTOR	RT-PCR, direct sequencing, immunohistochemistry
Sweeney et al. [6]	2014	United States	FFPE	28	*BRAF (13)*	V600E (12) and L597R (1)	MAPK/ERK	Targeted NGS panel and RNA sequencing, Sanger sequencing, immunohistochemistry, western blotting, SMO functional assays, and BRAF inhibitor studies
					*SMO (11)*	L412F (10)	Hedgehog	
					*SMO (11)*	W535L	Hedgehog	
					*KRAS (4)*	G12R	MAPK/ERK	
					*FGFR2 (5)*	C382R (4) and N549K	FGF/FGFR	
Oikawa et al. [41]	2013	Japan	FFPE, FT	18	*EGFR (0)*	No mutation	EGFR	Chromogenic *in situ* hybridization (CISH), Direct DNA sequencing, immunohistochemistry
Siriwardena et al. [42]	2009	Japan	FFPE	6	*CTNNB1 (0)*	No mutation	Wnt/β-catenin	Direct sequencing, immunohistochemistry
					*APC (3)*	G1339A	Wnt/β-catenin	
Tanahashi et al. [43]	2008	Japan	FFPE	18	*AXIN1 (1)*	Silent mutation	Wnt/β-catenin	Direct sequencing, immunohistochemistry
					*AXIN2 (1)*	SNP	Wnt/β-catenin	
Miyake et al. [44]	2006	Japan	FFPE	6	*CTNNB1 (1)*	T40I	Wnt/β-catenin	Direct sequencing, immunohistochemistry
Kawabata et al. [45]	2005	Japan		14	*CTNNB1 (1)*	N/A	Wnt/β-catenin	Direct sequencing
Kumamoto et al. [46]	2004	Japan	FFPE, FT	22	*KRAS (1)*	G12A	MAPK/ERK	Direct sequencing, immunohistochemistry
Kumamoto et al. [47]	2004	Japan	FFPE, FT	10	*TP53 (0)*	No mutation	p53	Direct sequencing, immunohistochemistry
Perdigão et al. [26]	2004	Brazil	FFPE, FT	4	*AMBN (5)*	One splice site mutation	N/A	Direct sequencing
					*AMBN (5)*	P81Q; T604A; M76R; Q54E	N/A	
Sekine et al. [48]	2003	Japan	FFPE	20	*CTNNB1 (1)*	S45P	Wnt/β-catenin	Direct sequencing and immunohistochemistry
Shibata et al. [49]	2002	Japan	FT	12	*TP53 (1)*	C238Y	p53	Yeast functional assay and direct sequencing

*N/A, Not Available*.

More than 25 different mutations were identified in ameloblastoma. *BRAF* was the most frequently affected gene. *BRAF* mutations were assessed in 23 studies, involving a total of 530 tumors. Approximately, 71% (*n* = 377) of the analyzed tumors harbored somatic mutations in *BRAF* being the V600E mutation the most commonly reported (Figure 3). Other single-nucleotide transversions affecting *BRAF* were also reported but were uncommon; BRAF T440P was found in two cases [25] and BRAF L597R in one case [6].

*SMO* was assessed in nine studies [6, 8–10, 13–16, 25] in which 264 tumors were evaluated trough Sanger sequencing, targeted NGS panels and WES. Somatic point mutations in *SMO* were present in 10.6% (*n* = 28) of the analyzed samples. L412F was the most common mutation found in 15 cases, followed by W535L in 4 cases and G416E in only one case. One article reported 6/42 cases to harbor mutations in exon 6 and 2/42 cases in exon 9 of the SMO gene [10]. Nevertheless, the authors did not specify more about those mutations (probably they are the aforementioned L412F and W535L, respectively). Three articles identified that *SMO mutations* were mutually exclusive with *BRAF mutations* [6, 10, 14], whereas others reported that *SMO mutations* co-occurred with background *BRAF* mutations [8]. Five articles did not identify any *SMO* mutations [9, 13, 15, 16, 25], either through WES [13, 15], targeted NGS panel [16, 25] or Sanger sequencing [9].

Mutations in other genes related to the mitogen-activated protein kinase (MAPK) pathway, such as *KRAS, NRAS, HRAS*, and *FGFR2* were identified in 4.3% (12/276) [6, 10, 12, 46], 3.5% (9/254) [10, 14, 16], 2.4% (6/254) [10, 14], and 5.5% (14/254) [6, 8, 12] of the analyzed samples, respectively (Table 3). These mutations tended to be mutually exclusive with *BRAF* mutations.

Mutations in the tumor suppressor gene *TP53*, were of low frequency reported only by two studies. Shibata et al. found *TP53* mutations in 1 of 12 ameloblastomas [49] and Bartels et al. in 1 of 7 [12]. Kumamoto et al., were unable to find *TP53* mutations in their cohort of 10 ameloblastomas [47]. Mutations in other tumor suppressor genes, such as *PTEN*, are also of low frequency *and* have been reported in 5/20 [34], 1/7 (12), and 2/62 ameloblastomas [10].

One article reported 45% of their cohort (9/20 ameloblastomas) to harbor missense mutations (in non-SNP sites) affecting *TSC1*. Correspondingly, those samples showed significantly lower mRNA expression levels compared to normal mucosa, suggesting a higher proliferation rate in ameloblastoma attributed to abnormal mTOR accumulation [40].

Two articles that performed WES reported the presence of mutations affecting *KMT2D* occurring in the background of *BRAF* mutations [13, 15]. Guan et al. [13], reported 2/10 ameloblastomas to harbor non-sense mutations in *KMT2D*, whereas Shi et al. [15], identified 1/4 ameloblastomas with a frameshift deletion in the same gene.

Odontogenesis-related genes have been widely associated with the etiopathogenesis of ameloblastoma. Somatic mutations in *BCOR* (inactivating frameshift deletion) *LRP6, SCN5A* (missense mutations in both), and *LAMB1* (frameshift deletion) were identified with WES [13], and co-occurred in the background of *BRAF* mutations. Three missense and one splicing site mutations affecting the ameloblastin gene (*AMBN*) were found in 4/4 ameloblastomas by direct sequencing [26]. Mutations related to Wnt/β-catenin pathway have been reported affecting *CTNNB1* in 3.3% (9/272) of the cases [8, 10, 13, 16, 42, 44, 45, 48]. Likewise, another member of this pathway, *APC*, was reported by one study to be mutated in 3/6 cases [42] and another study reported four different single nucleotide variations affecting different locus at this gene in a cohort of 30 patients, with a mutation rate that ranged from 6.25 to 27.5% [37] (Table 3). Contrary to this, Tanahashi et al., assessed *CTNNB1, APC, AXIN1*, and *AXIN2* in 18 ameloblastomas, and did not identify any missense mutations in these genes. However, the authors found one silent mutation in *AXIN1* and one single nucleotide polymorphism (SNP) in *AXIN2* [43]. Similarly, Siriwardena et al., did not identify mutations in *CTNNB1* among six ameloblastomas [42].

#### Chromosomal Alterations

Five articles reported chromosomal imbalances in ameloblastomas by using comparative genomic hybridization (CGH) [18, 19], microsatellite markers [27], WES [13] or whole-genome microarray [20]. Overall, these articles reported a relative stability in terms of chromosomal imbalances in ameloblastomas. Jääskeläinen et al., found copy number alterations (CNAs) in 2/17 ameloblastomas [18]; Toida et al., in 1/9 ameloblastomas [19]; whereas Diniz et al., reported seven rare CNAs (affecting 3 ameloblastomas) and 4 copy-neutral loss of heterozygosity (cnLOH) (affecting 2 ameloblastomas) [20]. Nodit et al., reported *L-myc* and *PTEN* as the two genes with most allelic losses (71 and 62%, respectively) and that the overal frequency of allelic loss was similar among ameloblastomas and ameloblastic carcinomas.

## Discussion

Odontogenic tumors (OT) arise from dental tissues or their remnants, and for decades, this statement was only supported by the histologic appearance of these lesions, which resembles the enamel organ, dental papilla or the dental follicle [1, 50, 51]. Increasing evidence showing mutations and/or chromosomal alterations in the same genes expressed during odontogenesis have confirmed this association, consolidating the close relationship between ontogenesis and oncogenesis [8, 9, 14, 16, 38, 52–60]. The ongoing development of the molecular aspects of odontogenic tumors has revolutionized the understanding of the ethiopathogenesis of these heterogenous group of lesions, allowing the proposal of novel molecular therapeutic targets. However, the exact mechanism underlying the tumorigenic process and possible causes for their different clinical behavior remains unknown.

Both AOT and AM are odontogenic tumors of epithelial origin, but their clinical behavior is diametrically different, resulting in different treatment approaches and prognosis. In the current systematic review, we aimed to compare the genetic alterations of AOT with the ones reported in AM, in order to summarize the current genetic knowledge of these lesions and aid in the understanding of the genomic alterations underlying their development and different behavior.

Our search identified six studies that analyzed the genetic aspects of AOTs (*n* = 59), in contrast to 37 that explored the genetic landscape of ameloblastoma (*n* = 530). Mutation in exon 12 of *KRAS* was found to be present in 76% of AOTs with G12V/R being the most found [22, 23]. On the other hand, mutations in *BRAF* were found in 71.1% of the samples, corresponding mainly to V600E [6, 25] (Figure 3). Interestingly, the proportion of AOTs with *KRAS* driver mutation, is similar to the proportion of driver mutations reported in ameloblastoma. Due to its high frequency, *KRAS* mutations were proposed as a driver mutation and signature marker of AOTs [22–24]. Because *KRAS* mutations are a recurrent finding in AOTs, Coura et al. [22], suggested the presence of KRAS G12V/R to help in the diagnosis of controversial cases of AOT, in the same way that BRAF V600E could be used in routine ameloblastoma diagnostics [14, 38].

The RAS oncogene family is comprised of three members, *KRAS, NRAS*, and *HRAS*, and plays an important role in normal development, but also for cancer development. Activated point mutations on RAS proteins are widely present across a different spectrum of human cancers [61, 62]. Our review showed that all *KRAS* mutations reported in AOTs have been found affecting codon 12 [22–24]. Mutations affecting this codon have been reported in non-small cell lung cancer and pancreatic ductal adenocarcinoma, being present in almost half of the cases for the former, and in 16% for the latter [63, 64]. KRAS corresponds to a small GTPase that transduces extracellular signals to intracellular signal transduction cascades [65] (Figure 3). It has been suggested that the mutation subtype may affect downstream signaling differently, which could be reflected clinically [66, 67]. Nevertheless, to date, this has not been demonstrated in AOTs. Coura et al., reported in their cohort of 38 AOTs, no statistically significant association between the presence of mutations (mainly KRAS G12V and G12R) and clinicopathological parameters (including patient's age, tumor size, location, follicular or extrafollicular variants, and fibrous capsule thickness) [22].

The activation of RAS/GTP complexes, can activate several downstream signaling pathways such as Raf-MEK-ERK, PI3K-AKT-mTOR, RalGDS-RalA/B, and the TIAM1-RAC1 [65]. To date, only the activation of the MAPK/ERK pathway has been demonstrated in AOT. With immunohistochemistry, Coura et al., demonstrated not only *KRAS*-mutated cases, but also wild-type *KRAS* cases to have strong pERK1/2 expression. This suggests that the MAPK pathway can be activated by other mechanisms rather than *KRAS* mutations [22]. Similarly, using immunohistochemical techniques, Bologna-Molina et al., demonstrated AOTs to express different proteins related to the MAPK/ERK pathway, including EGFR, KRAS, BRAF, CRAF, ERK, and MEK [24].

Apart from *KRAS* mutations, other somatic point mutations affecting *SMO* and *AMBN* [25, 26], and losses affecting 7p15.3 and 6q15 [23], were also found in AOTs. In a similar way, other somatic mutations have been reported in ameloblastoma, mainly affecting: *SMO* [6, 8, 10, 14], other MAPK pathway-related genes such as *KRAS, NRAS, HRAS, FGFR2* [6, 8, 10, 12, 14, 16] and in a lower frequency, *PTEN* [10, 12, 34] and *CTNNB1* [8, 10, 12, 13, 16, 34, 42, 44, 45] among others. Interesting results were found by Diniz et al., who reported one ameloblastoma negative for BRAFV600E, with greater number of CNAs and cnLOH encompassing genes directly related with RAF/MAPK pathway activation, suggesting an alternative mechanism of mimicking this pathway [20].

Recently, Bello et al. [68] proposed that the interactions between the adhesion proteins FAK, paxillin and PI3K may be relevant in the aggressiveness of AM compared to AOT, based on the observation that FAK expression was stronger in AM compared to AOT, and that one case of peripheral AM with strong expression of the three proteins had a history of two recurrences. Nevertheless, their conclusions should be carefully interpreted because were based on observations based on a small cohort of AOTs (*n* = 7).

The biological nature of AOT has been a constant matter of debate. In 2017, Reichart et al. [69], compared the immunohistochemical expression of different factors between AOT and AM, and proposed AOT to be a hamartomatous process rather than a true neoplasm [69]. Markers related to invasion, such as cytokeratin profiles and integrins, to proliferation, such as MDM2, p53 protein and metallothionein levels, were found to be higher in ameloblastomas compared to AOTs. Also, AOTs showed lower levels of matrix metalloproteinases (consistent with a reduced local aggressiveness), Ki76 and anti-apoptosis markers such as Bcl-2, and higher levels of β-catenin (suggesting greater cell adhesion properties) [69]. Similarly, there are publications about the strong cytoplasmatic expression of β-catenin on AOTs [4, 21], however no mutation in *CTNNB1* was detected [4]. The proposal of Reichart et al. [69] was based purely on immunohistochemical findings without considering genetic aspect. Although our knowledge about the molecular background of AOT is still very limited, the genetic data collected by this review points to the direction that AOT harbor mutations in important oncogenic driver genes, such as KRAS, and based purely on this, some authors have proposed it as a neoplasm [22]. Nevertheless, until now, the presence of these genetic alteration seems not to have a direct impact on its clinical behavior. Thus, care has to be taken when interpreting these findings.

The low number of studies that have performed small to large-scale and/or “omics” techniques to characterize the molecular background of AOTs, the low frequency of AOT (accounting for <5% of odontogenic tumors) [70–72], limited clinical information availability, and the fact that most of the available studies come from single-institution series or case reports, limit the conclusions than can be drawed out of these findings. Also, current publications are all retrospective studies based on formalin-fixed paraffin-embedded samples (much of them subjected to decalcifications methods), which shows inherent limitations, mainly related to the quality of the nucleic acids for these purposes and the difficulty of retrieving a large cohort. Nevertheless, molecular pathology is demonstrating its utility in the diagnosis of challenging cases and for targeted therapy of disfiguring tumors such as ameloblastoma, avoiding considerable post-surgical morbidities.

## Conclusions

The available genetic data reports that 75% of AOTs harbor somatic mutations in *KRAS*, a well-known oncogene. Nevertheless, the number of studies that have a assessed the genetic landscape of AOT is still very limited, not providing enough evidence to draw a conclusion regarding the relationship between the genomic alterations and its clinical behavior. There are a significant number of studies that have assessed the genetic aspects of ameloblastoma. Different genetic alterations have been reported, being the BRAFV600E mutation the most common. The relatively high frequency of ameloblastoma compared to other odontogenic tumors, such as AOT, has facilitated the performance of different sequencing techniques, allowing the discovery of different mutational signatures. On the contrary, the low frequency of AOTs is an important limitation for this. Thus, the presence of other mutational signatures with clinical impact, co-occurring with *KRAS* background or in wild-type *KRAS* cases, cannot be ruled out.

Since BRAF and RAS are in the same MAPK pathway, it is interesting that ameloblastomas, frequently associated with BRAFV600E mutation have aggressive clinical behavior, but in contrast, AOTs, frequently associated with RAS mutations have indolent behavior. Functional studies might be required to solve this question.

## Data Availability Statement

The original contributions presented in the study are included in the article/supplementary material, further inquiries can be directed to the corresponding author.

## Author Contributions

All authors listed have made a substantial, direct and intellectual contribution to the work, and approved it for publication.

## Conflict of Interest

The authors declare that the research was conducted in the absence of any commercial or financial relationships that could be construed as a potential conflict of interest.

## Publisher's Note

All claims expressed in this article are solely those of the authors and do not necessarily represent those of their affiliated organizations, or those of the publisher, the editors and the reviewers. Any product that may be evaluated in this article, or claim that may be made by its manufacturer, is not guaranteed or endorsed by the publisher.
